# Shape Shifting: Local Landmarks Interfere With Navigation by, and Recognition of, Global Shape

**DOI:** 10.1037/a0034901

**Published:** 2013-11-18

**Authors:** Matthew G. Buckley, Alastair D. Smith, Mark Haselgrove

**Affiliations:** 1 School of Psychology, University of Nottingham, Nottingham, England

**Keywords:** geometric module, associative learning, spatial learning, navigation, ID-ED

## Abstract

An influential theory of spatial navigation states that the boundary shape of an environment is preferentially encoded over and above other spatial cues, such that it is impervious to interference from alternative sources of information. We explored this claim with 3 intradimensional–extradimensional shift experiments, designed to examine the interaction of landmark and geometric features of the environment in a virtual navigation task. In Experiments 1 and 2, participants were first required to find a hidden goal using information provided by the shape of the arena or landmarks integrated into the arena boundary (Experiment 1) or within the arena itself (Experiment 2). Participants were then transferred to a different-shaped arena that contained novel landmarks and were again required to find a hidden goal. In both experiments, participants who were navigating on the basis of cues that were from the same dimension that was previously relevant (intradimensional shift) learned to find the goal significantly faster than participants who were navigating on the basis of cues that were from a dimension that was previously irrelevant (extradimensional shift). This suggests that shape information does not hold special status when learning about an environment. Experiment 3 replicated Experiment 2 and also assessed participants’ recognition of the global shape of the navigated arenas. Recognition was attenuated when landmarks were relevant to navigation throughout the experiment. The results of these experiments are discussed in terms of associative and non-associative theories of spatial learning.

The ability to learn the location of significant objects within the environment is an integral part of life for both human and non-human animals. For non-human animals, this ability permits the distinction of locations that signal the presence of food, water, shelter, or safety from prey. For humans, this ability permits us to travel back and forth between locations in a multitude of environments, both real (e.g., home, work, the shops) and virtual (e.g., in computer games). Studies have shown that a plethora of stimuli can be used to aid navigation, which include the shape or boundaries of an environment (e.g., Pearce, Ward-Robinson, Good, Fussell, & Aydin, 2001), landmarks that are both distal and proximal to a goal location (Prados, Redhead, & Pearce, 1999; A. D. L. Roberts & Pearce, 1998; Save & Poucet, 2000), the slope of the floor (Nardi & Bingman, 2009; Nardi, Newcombe, & Shipley, 2011; Nardi, Nitsch, & Bingman, 2010), as well as internally derived cues such as vestibular (e.g., Wallace, Hines, Pellis, & Whishaw, 2002) and somesthetic information (Lavenex & Lavenex, 2010).

Many experiments have now demonstrated that learning to navigate toward a goal by using landmarks can be influenced by the presence of other environmental cues. For example, Chamizo, Manteiga, Rodrigo, and Mackintosh (2006) demonstrated that rats’ ability to use a distal landmark to find a hidden goal was restricted if another landmark, more proximal to the goal, was co-present during the training trials (see also Chamizo, Aznar-Casanova, & Artigas, 2003; Gould-Beierle & Kamil, 1999; Leising, Garlick, & Blaisdell, 2011; A. D. L. Roberts & Pearce, 1999; Sanchez-Moreno, Rodrigo, Chamizo, & Mackintosh, 1999; Stahlman & Blaisdell, 2009). The ability of one cue to restrict, or interfere with, learning about another cue has also been demonstrated on numerous occasions in studies of classical conditioning using a variety of procedures (e.g., Jones & Haselgrove, 2011; Pavlov, 1927) and has led to the suggestion that learning to navigate is underpinned by a general, associative, mechanism that is also responsible for learning in other, non-spatial domains (Pearce, 2009). This suggestion has, however, not gone unchallenged, as other authors have emphasized the special status of information provided by the *shape*, or *geometry*, of an environment for navigation (for reviews, see Cheng, 2008; Jeffery, 2010; Lew, 2011; Pearce, 2009). Cheng (1986) proposed that geometric information is processed in a dedicated module that is impervious to the influence of learning about landmark cues, and this position was vehemently championed by Gallistel (1990), who, in the context of discussing Cheng’s ideas and data, suggested the following:

this organ [the geometric module] constitutes a module in Fodor’s (1983) sense; it works only with certain kinds of information, even under circumstances where other kinds of readily perceptible data are highly relevant to successful performance. Fodor termed this property of the module impenetrability. (p. 208)

Doeller and Burgess (2008; see also Barry et al., 2006; Burgess, 2006, 2008; Cheng & Newcombe, 2005; Wang & Spelke, 2002; White & McDonald, 2002) have more recently suggested a notion reminiscent of the geometric module. They proposed that while landmark learning may obey the rules of associative learning (e.g., Mackintosh, 1975; Pearce & Hall, 1980; Rescorla & Wagner, 1972), “learning relative to environmental boundaries is incidental, occurring independently of behavioral error or the presence of other predictive cues” (Doeller & Burgess, 2008, p. 5912; see also Burgess, 2006, 2008).

The converging prediction from these theories, which suggest that geometric/boundary information is processed in a modular fashion, is that landmarks should not interfere with learning about the shape of the environment, and, indeed, several studies support this contention. Cheng (1986) trained rats to find food that was hidden in one corner of a rectangular arena, which contained a distinctive landmark in each corner. In order to find the food, rats could rely on the geometric information provided by the rectangular arena or on the unique landmarks located in each corner of the rectangle. Relying on the geometric information provided by the rectangular arena would lead rats to search in either the correct corner, or the diametrically opposite corner that is geometrically identical to the correct corner. The four unique landmarks, however, disambiguated all the corners of the rectangle from each other; thus, relying on the unique landmarks would lead rats to search only in the correct corner. When the landmarks were removed from the corners of the rectangle, rats searched in both the correct and diametrically opposite corners more often than in the remaining two corners. Thus, the presence of the more predictive landmark cues did not preclude learning that was based upon the less predictive geometry of the rectangle. Similar effects have frequently been demonstrated in other experiments with rats (Graham, Good, McGregor, & Pearce, 2006; Hayward, Good, & Pearce, 2004; Hayward, McGregor, Good, & Pearce, 2003; McGregor, Horne, Esber, & Pearce, 2009; Pearce et al., 2001; Wall, Botly, Black, & Shettleworth, 2004) as well as pigeons (Kelly, Spetch, & Heth, 1998), and with humans navigating in virtual environments (Doeller & Burgess, 2008; Redhead & Hamilton, 2007, 2009). There are, however, a number of reports of landmark cues interfering with learning about geometric information. For example, in an overshadowing experiment by Pearce, Graham, Good, Jones, and McGregor (2006), an experimental group of rats was trained to find a goal that was hidden in one corner of a rectangular arena consisting of two long black walls and two short white walls. Relying on the geometry or the wall colors of each corner would lead the rats to the correct or the geometrically equivalent corner of the rectangle. For a control group, the color of the short and long walls changed, randomly, between trials; thus, only geometric information would permit navigation to the correct, or geometrically equivalent, corner. In a test trial conducted in an all-white rectangle, the control group spent significantly longer than the overshadowing group searching in the correct or geometrically equivalent corners. The clear implication of these results is that learning about geometric information can be modulated by non-geometric information (see also Cole, Gibson, Pollack, & Yates, 2011; Horne, Iordanova, & Pearce, 2010; Horne & Pearce, 2009, 2011; Pearce et al., 2006; Prados, 2011; Redhead & Hamilton, 2009; Wilson & Alexander, 2008).

In order to provide a reconciliation of these conflicting results, Miller and Shettleworth (2007, 2008) suggested an associative analysis of spatial navigation that does not make the assumption that learning about boundary geometry is impervious to interference by landmarks. Briefly, they suggested that the geometric information conveyed by the corners of an environment and landmarks, either within or outside the environment’s boundaries, is encoded as representational elements. These elements can compete for an association with the navigational goal (e.g., a platform in the case of a water maze) according to a modification of the learning rule proposed by Rescorla and Wagner (1972):

$$\Delta {\text{V}}_{\text{E}}=\;\alpha \left(\lambda -{\text{V}}_{\text{L}}\right)P_{\text{L}}$$ 1

Here, V_E_ is the strength of the association between a representational element and the navigational goal, α is the inherent (i.e., not modifiable) salience of the element, λ is the asymptote of learning supported by the goal (1 when it present, 0 when it is not), V_L_ is the associative strength of all elements at a particular location, and *P*_L_ is the probability of choosing a particular location, which itself is defined as:

$$P_{\text{L}}={\text{V}}_{\text{L}}/\Sigma {\text{V}}_{\text{L}}$$ 2

Where, finally, ΣV_L_ is the sum of the associative strengths of all locations. Incorporating *P*_L_ into the learning equation permits Miller and Shettleworth (2007, 2008) to predict that the presence of a landmark will—under some circumstances—restrict learning about the geometry of an environment (i.e., overshadow it) but under other circumstances will not. Consider the case in which a navigational goal is located in one corner of a rectangular arena which contains no landmarks. Equation 1 ensures that the geometric elements of the correct corner will become associated with the navigational goal. However, this learning will progress relatively slowly because the diametrically opposite, geometrically equivalent, corner will occasionally be visited, and the goal will not be present—fostering extinction of the association between these elements and the goal. Now consider the case of an “overshadowing” group who again has a navigational goal located in one corner of a rectangular arena but also has placed within that corner a distinctive landmark. Equation 1 ensures that the association between the geometric elements within the correct corner and the navigational goal will increase and, correspondingly, so too will the probability of visiting this corner. However, the geometrically equivalent corner, which contains neither the goal nor a landmark, will not be visited so frequently—as it is not identical to the correct corner. Consequently, the elements shared by the correct and geometrically equivalent corners will tend to gain, but not lose, associative strength (as the geometrically equivalent corner will be visited only infrequently). Relative to a control group, therefore, early in training the presence of a landmark might actually serve to enhance learning about the geometry of an environment undermining the overshadowing effect, which should eventually be observed with sufficient training. With certain parameters granted, Miller and Shettleworth (2007) were able to successfully simulate studies in which landmarks have successfully overshadowed learning about boundary geometry and, indeed, studies in which landmarks failed to overshadow learning about boundary geometry.

By adopting and adapting the Rescorla–Wagner learning algorithm, the theory described by Miller and Shettleworth (2007, 2008) provides a compellingly simple explanation for both the presence and absence of cue-competition between navigational features. However, by using the Rescorla–Wagner model as its starting point, the Miller–Shettleworth analysis of spatial navigation also inherits a number of its limitations. One particular limitation is the assumption that the salience of representational elements (α) is fixed; this assumption precludes the Rescorla–Wagner model, and hence the model proposed by Miller and Shettleworth, from explaining the intradimensional–extradimensional (ID-ED) shift effect. The simple form of an ID-ED experiment comprises two stages of training and a set of stimuli drawn from two different dimensions (Mackintosh & Little, 1969). In the first stage, participants are trained that stimuli from one dimension are relevant to acquiring the outcome of the trial, while those from a second dimension are irrelevant. During the second stage of the experiment, novel stimuli from the dimensions used in Stage 1 are presented. For participants undergoing an intradimensional (ID) shift, the same dimension remains relevant for the solution of the task, whereas for participants undergoing an extradimensional (ED) shift, the previously irrelevant dimension becomes relevant. For example, Trobalon, Miguelez, McLaren, and Mackintosh (2003) employed an ID-ED procedure in a spatial navigation task in which rats in an ID group received food when they visited the western, but not the northern, arm of a radial maze. Rats in an ED group received food when they visited an arm of the maze textured with wood but not plastic. In Stage 2 of the experiment, all rats were rewarded for running down the south-west arm, but not the south-east arm, of the same maze. The results indicated that rats in the ID group solved the task more readily in Stage 2 than did the rats in the ED group. In their discussion of the ID-ED effect, both Mackintosh (1975, p. 279) and Le Pelley (2004, p. 212) argued that the observed retardation of learning in ED groups relative to ID groups, during the second discrimination, can only be explained by variations in the attention paid (α) to relevant or irrelevant stimuli. On this basis, therefore, it seems that Miller and Shettleworth’s theory is unable to provide an explanation for Trobalon et al.’s demonstration of an ID-ED effect within the spatial domain. However, the theory provided by Miller and Shettleworth focused on how learning about spatial features (such as landmarks) interacts with geometry learning, and to date there exists no study that has examined whether the ID-ED effect persists in spatial navigation when landmarks and boundary geometry are manipulated in such a way. This is somewhat surprising because, as the name implies, the ID-ED shift procedure examines the effect of shifting either within or between different categories of stimuli (dimensions) on learning a task. The procedure is therefore intrinsically suited to addressing the interaction between landmarks and geometry in spatial navigation, yet, surprisingly, the ID-ED procedure has not been applied to this question.

As the ID-ED shift procedure establishes one dimension as entirely irrelevant to the purpose of acquiring the goal, or outcome of a task, and a second dimension as fully predictive of the goal, the procedure is also ideal for testing the claims of Gallistel (1990) and Doeller and Burgess (2008), who have suggested that learning about the geometry, or boundary, of an environment will be unaffected by other highly relevant data, or predictive cues. According to these analyses, even if the geometry/boundary of an environment is established as entirely irrelevant (and other cues as fully predictive) for navigation in Stage 1 of the experiment, subsequent navigation based upon geometry/boundaries in Stage 2 should be entirely unaffected. If this result were obtained, it would constitute particularly strong evidence for the modular basis of geometry in spatial navigation. In contrast, should the current experiments demonstrate superior learning in participants undergoing an ID, rather than an ED, shift then the modular analysis of geometry in navigation will be undermined. Furthermore, should an ID-ED effect be observed, it will be possible to make a more constrained theoretical interpretation of how landmarks and boundary geometry interact, as the ID-ED effect is widely acknowledged to indicate the effect of learned attentional changes to cues (e.g., Esber & Haselgrove, 2011; Le Pelley, 2004; Mackintosh, 1975; for a related point, see Le Pelley, Schmidt-Hansen, Harris, Lunter, & Morris, 2010). To avoid undue repetition, we restrict our discussion of these theories to the general discussion.

In the three experiments reported here, human participants were first trained that either landmarks or the geometric properties of the boundary of a distinctively shaped arena were relevant to finding a hidden goal in Stage 1. In Stage 2, novel landmarks were presented in an arena of a different shape and participants completed either an ID or an ED shift from Stage 1. According to theories which suggest that learning about geometric information does not interact with learning about landmarks during navigation (e.g., Cheng, 1986), as well as Miller and Shettleworth’s (2007, 2008) associative theory, performing an ED shift should have no effect on performance relative to the ID group. A slightly different pattern of predictions can be derived from the analysis of spatial navigation provided by Doeller and Burgess (2008). If learning about the boundaries of an environment occurs independently of behavioral error and is not prone to interference from learning about landmarks, then training that establishes the shape of an environment as irrelevant to finding the goal in Stage 1 should not retard subsequent learning about the boundary shape in Stage 2, at least relative to training in which the shape of an environment was not irrelevant in Stage 1. In contrast, if landmark learning obeys general associative learning principles and is prone to interference from learning about boundary information, then training that establishes landmarks as irrelevant to finding the goal in Stage 1 would be expected to produce retarded learning about landmarks in Stage 2, again, relative to training in which landmarks has never been irrelevant.

## Experiment 1

In Stage 1, participants were trained to find a hidden goal that, on each trial, was always located in one of the four corners of a kite-shaped virtual arena, each of which was colored a different shade of blue. We refer to the landmarks created by the shading of the corners of the walls as *wall panels*. The positions of these wall panels changed to different corners on each trial. For half the participants in Stage 1, the hidden goal could only be located with reference to information provided by the shape of the arena; thus, information provided by the landmarks was irrelevant. For example, the goal might always be hidden at the most acute corner of the kite—the color of which changed on a trial by trial basis. For the remainder of the participants, the hidden goal could only be located with reference to one of the four wall panels; information provided by the shape of the arena was irrelevant to finding its specific location. For example, the goal might always be hidden in the corner that was the darkest shade of blue—irrespective of which corner this shade was located. In Stage 2 of the experiment, participants had to learn to find a hidden goal in a trapezium-shaped arena, the corners of which were four different shades of red. As before, the positions of the landmarks changed to different corners on each trial. During Stage 2, participants who completed an ID shift had to learn about a cue from the same dimension that was relevant to finding the goal in Stage 1. Thus, if the shape of the arena was relevant to finding the goal in Stage 1, then it was also relevant to finding the goal in Stage 2 (group Shape–Shape). Likewise, if landmarks were relevant to finding the hidden goal in Stage 1, then they were also relevant to finding the goal in Stage 2 (group Landmark–Landmark). Participants who completed an ED shift, however, had to learn in Stage 2 about a cue from the dimension that was irrelevant to finding the goal in Stage 1. Consequently, participants who had learned the location of the goal with respect to the shape of the arena in Stage 1 had to learn the location of the goal with respect to landmarks in Stage 2 (group Shape–Landmark), and participants who had learned the location of the goal with respect to landmarks in Stage 1 had to learn the location of the goal with respect to the shape of the arena in Stage 2 (group Landmark–Shape). To assess navigational behavior over the course of the experiment, both the time taken and the distance traversed to find the hidden goal were recorded on each trial. The latency to find the goal is a common measure in studies of spatial navigation both in animals (e.g., Morris, 1981; Pearce, Roberts, & Good, 1998) and humans (e.g., Wilson & Alexander, 2008), and path length measurements are also common in both animal (e.g., Bast, Wilson, Witter, & Morris, 2009) and human (e.g., Redhead & Hamilton, 2007) experiments.

### Method

#### Participants

Forty-eight participants were recruited from the University of Nottingham (31 females). Participants were randomly allocated to one of the four groups in the experiment and were given course credit or £5 in return for participation. The age of participants ranged from 18 to 28 years (*M* = 19.31, *SEM* = 0.27). An additional £10 was awarded to the participant who completed the experiment in the shortest time.

#### Materials

**Figure 1 fig1:**
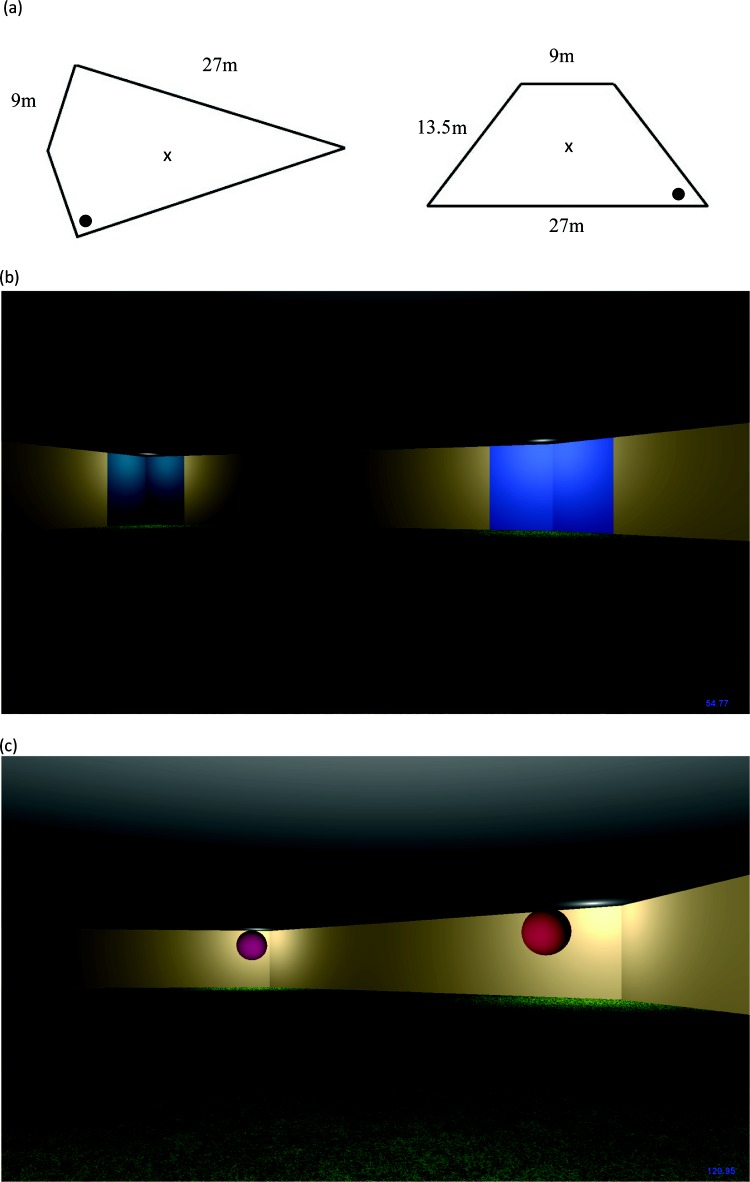
(a) Plan views of the two arenas, with apparent wall length indicated. Circles represent one of the four possible goal locations in each arena; “x” represents the starting location of participants. (b) Screen shot of an example of the kite-shaped arena used in Experiment 1. (c) Screen shot of an example of the trapezium-shaped arena used in Experiments 2 and 3.

All virtual environments were constructed, complied, and displayed using Mazesuite software (Ayaz, Allen, Platek, & Onaral, 2008; www.mazesuite.com), which were run on a standard Stone desktop computer, running Microsoft Windows 7. A large Mitsubishi LDT422V LCD screen (935 × 527 mm) was used to display the virtual environments. All virtual arenas were viewed from a first-person perspective, and a grass texture was applied to the floor of each arena. Using the 0–255 RGB scale employed by Mazesuite, the cream-colored walls in the kite and trapezium were defined as 204, 178, 127. Assuming a walking speed similar to that in the real world (2 m/s), the perimeter of the kite was 72 m, with the small walls being 9 m in length and the long walls 27 m. The height of the arenas was approximately 2.5 m. The kite was configured such that it contained two right angles corners with the remaining two angles being 143.14° and 36.86°, and the isosceles trapezium contained angles of 48.19° and 131.81°. The perimeter of the trapezium was 63 m, with the smallest wall being 9 m, the largest wall 27 m, and the remaining two walls 13.5 m in length (see Figure 1a).[Fig-anchor fig1]

Four pairs of colored wall panels, each 1.13 m in length and approximately 2.5m in height, served as landmarks and were located on either side of each corner in an arena. The four blue wall panels presented in the kite-shaped arena were defined as RGB: 25, 127, 102; 25, 102, 127; 0, 25, 102; and 51, 102, 204; and the four red wall panels presented in the trapezium-shaped arena were 127, 25, 51; 127, 51, 76; 10, 25, 102; and 51, 25, 76. The goals within the arenas were square shaped regions (1.08 m × 1.08 m, invisible to participants) that were always located 1.475 m away from the walls of the arena, along on a notional line that bisected the corner in half. A third arena was also utilized in this experiment, which was designed to allow participants to become familiar with the controls of the experimental task. This exploration arena was a regular octagon configured of red walls (RGB: 229, 25, 51), with a grass texture again applied to the floor. There was no hidden goal present. Again assuming a walking speed of 2 m/s, each wall was of the exploration arena was 12 m in length. Figure 1b shows a screen shot of an example of the kite-shaped arena used in Experiment 1.

#### Procedure

After signing a standard consent form, participants were given the following set of instructions on paper:

This study is assessing human navigation using a computer generated virtual environment. During this experiment, you will complete 48 trials. In each trial, you will be placed into a room that contains an invisible column. Your aim is to end the trials as quickly as possible by walking into the column.

You will view the environment from a first person perspective, and be able to walk into the column from any direction using the cursor keys on the keyboard. Once you’ve found the column a congratulatory message will be displayed and you should hit enter when you’re ready to begin the next trial. You will always be in the centre of the arena when a trial begins, but the direction in which you face at the start of each trial will change.

To start with, you may find the column is difficult to find. There is, however, a way of learning exactly where the invisible column will be on each trial. It’s a good idea to fully explore the environment on the first few trials, this will help you to learn where the column is going to be.

This session should take around 30–40 minutes. If at any point you wish to stop this session, please notify the experimenter and you’ll be free to leave without having to give a reason why. Your results will be saved under an anonymous code, and kept confidential throughout.

The person who takes the least time to complete this experiment will win a £10 prize!

Participants were sat not more than 100 cm from the screen, and first provided with the opportunity to move around the octagonal exploration arena for two 30-s trials using the four keyboard cursor keys. Presses on the “up” and “down” cursor keys permitted the participant to move forward and backward within the arena, respectively. Presses on the “left” and “right” cursor keys permitted the participant to rotate counter-clockwise and clockwise within the arena, again, respectively. Following these exploration trials, participants pressed enter to begin the first experimental trial. In the kite-shaped arena, participants began each trial at a point located halfway between the acute and obtuse corners, and in the trapezium shaped arena at a point half way along a notional line from the center of the shortest wall to the center of the longest wall. The direction in which participants faced at the start of each trial was randomized for every trial. Generating every possible configuration of four landmarks in the four corners of the arenas produced 24 different trials for both the kite- and the trapezium-shaped arenas. Each of these arenas was presented once to each participant, the order of which was randomized for each participant independently. Participants were first required to complete 24 trials in the kite shaped arena (Stage 1), before completing 24 trials in the trapezium shaped arena (Stage 2). On each trial, participants were required to find the hidden goal by using the four cursor keys as described above. There was no time limit for any trials; thus, each trial ended only when the hidden goal was found. Once the hidden goal had been found, participants could no longer move within the arena, and a congratulatory message (“Congratulations, you found the goal!”) was displayed on screen using the default font and character size in Mazesuite. Participants pressed enter to begin the next trial.

During Stage 1 for participants in groups Shape–Shape and Shape–Landmark, and during Stage 2 for participants in groups Shape–Shape and Landmark–Shape, the goal was located in the same corner of the arena on each trial. Each of the four wall panels was located in the goal corner on six trials, and in non-goal locations on the remaining 18 trials. During Stage 1 for participants in groups Landmark–Landmark and Landmark–Shape, and during Stage 2 for participants in groups Landmark–Landmark and Shape–Landmark, the goal was located adjacent to the same wall panel on each trial. Each of the four corners contained the goal on six trials and did not contain the goal on the remaining 18 trials.

Full details of Stage 1 and Stage 2 counterbalancing are given in the Appendix. We draw the reader’s attention, however, to the counterbalancing employed for group Shape–Shape, which was arranged such that any direct transfer of local geometric cues from the kite to the trapezium would not aid performance. For instance, if the goal in the kite was located in a corner where the right hand wall was long and the left hand wall short, the goal position in the trapezium would always be located where the left hand wall was longer than the right hand wall. Similarly, if the goal location in the kite was in the acute or obtuse angled corners, then in the trapezium the goal would be located in an obtuse or acute angled corner, respectively.

### Results and Discussion

**Figure 2 fig2:**
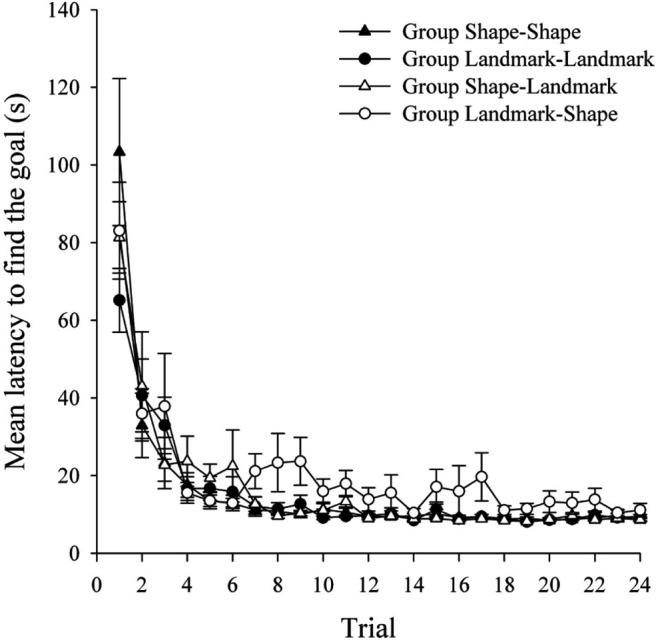
Mean latencies of the four groups to find the hidden goal in Stage 1 of Experiment 1. Error bars show 1 ± *SEM*.

An alpha value of .05 was adopted for all statistical tests in this and the following experiments. Figure 2 shows the latency, in seconds, from the beginning of each trial to enter the region defined as the hidden goal for the four groups during the 24 trials of Stage 1 of the experiment. The mean latencies in the four groups decreased across this stage of the experiment, but there was little indication of any differences between the groups. A two-way analysis of variance (ANOVA) of individual latencies, with the variables of relevant cue in Stage 1 (landmarks or shape) and trial (1–24), revealed a significant main effect of trial, *F*(23, 1058) = 55.55, *MSE* = 212.55, reflecting that the latency to find the goal decreased over trials. There was no main effect of relevant cue, *F*(1, 46) = 1.05, *MSE* = 751.87; however, there was a significant Trial × Relevant Cue interaction, *F*(23, 1058) = 1.72, *MSE* = 212.55. Simple main effects analysis revealed shorter latencies to find the goal in the landmark- than in the shape-relevant groups on Trial 1 but the reverse pattern on Trial 3, *F*s(1, 1104) > 7.86, *MSE* = 235.02. However, no significant differences in performance were noted by the end of Stage 1.[Fig-anchor fig2]

**Figure 3 fig3:**
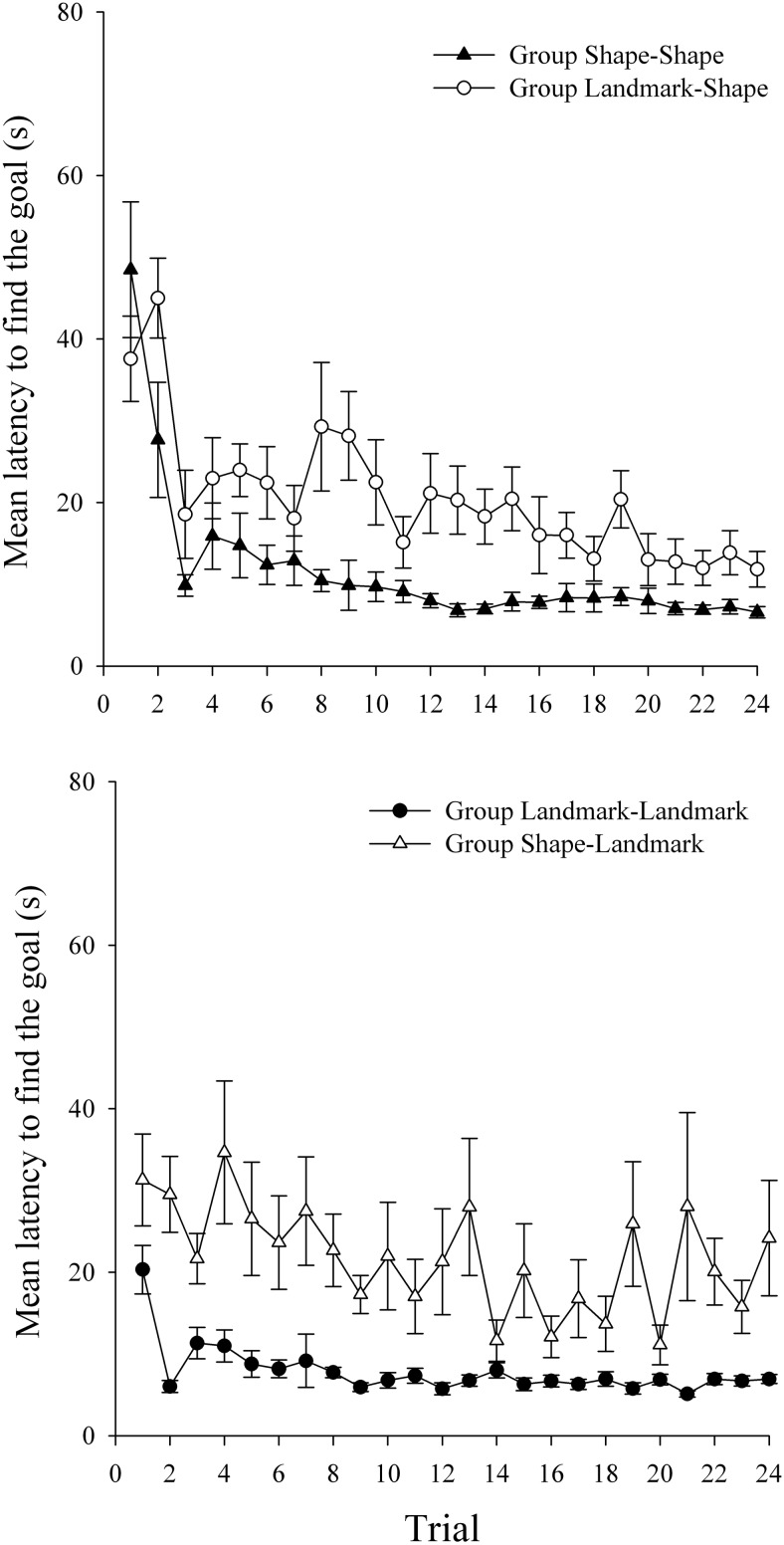
Top panel: mean latencies of groups Shape–Shape and Landmark–Shape to find the hidden goal in Stage 2 of Experiment 1. Bottom panel: mean latencies of groups Shape–Landmark and Landmark–Landmark to find the hidden goal in Stage 2 of Experiment 1. Error bars show 1 ± *SEM*.

The mean latency to find the goal during Stage 2 are shown in the top panel of Figure 3 for groups Shape–Shape and Landmark–Shape, and in the bottom panel of Figure 3 for groups Landmark–Landmark and Shape–Landmark. It can be seen that both of the groups that performed an ED shift (groups Landmark–Shape and Shape–Landmark) had longer latencies to find the goal relative to the appropriate ID groups (groups Shape–Shape and Landmark–Landmark). A three-way ANOVA of individual latencies to find the goal, with the variables of shift (ID or ED), relevant cue in Stage 2 (shape or landmarks), and trial (1–24), revealed a significant main effect of trial, *F*(23, 1012) = 9.65, *MSE* = 155.94; of shift, *F*(1, 44) = 43.12, *MSE* = 871.22; but no effect of relevant cue, *F* < 1. Crucially, there was a significant Shift × Trial interaction, *F*(23, 1012) = 1.71, *MSE* = 155.94. Simple main effects analysis of this interaction revealed that the ED shift groups, overall, were significantly slower to find the goal than the ID shift groups on Trials 2–13, 15, 17, 19, and 21–24, *F*s(1, 1056) > 3.961, *MSE* = 185.75. There was no Shift × Relevant Cue interaction, *F*(1, 44) = 2.12, *MSE* = 871.22; however, there was a Relevant Cue × Trial interaction, *F*(23, 1012) = 2.84, *MSE* = 155.94. Simple main effects analysis revealed that participants who were navigating on the basis of landmarks were significantly quicker at finding the goal on Trials 1 and 2 than participants navigating in the basis of shape, *F*s(1, 1056) > 19.16, *MSE* = 185.75. The three-way interaction was not significant, *F*(23, 1012) = 1.06, *MSE* = 155.94.[Fig-anchor fig3]

Analysis of path length data can be found in the online supplemental materials accompanying this article. For the sake of brevity, it is sufficient, here, to note that a three-way ANOVA of individual distances traversed, with the variables of shift (ID or ED), relevant cue in Stage 2 (shape or landmarks), and trial (1–24), revealed that the interaction between shift and trial was significant, *F*(23, 1012) = 1.76, *MSE* = 540.56. Simple main effects analysis revealed that the ED groups traveled a greater distance to find the goal than the ID groups on Trials 2–10, 12–13, 15, 17, 19, 21–22, and 24, *F*s(1, 1056) > 4.45, *MSE*s = 592.38.

Establishing either landmarks or the geometry of the environment as relevant to navigation influences the speed at which novel stimuli drawn from these stimulus categories are subsequently learned about. Specifically, (1) when landmarks have successfully guided navigation in the past, then subsequent navigation using information provided by the geometry of the arena is retarded relative to a group who initially navigated using geometry; and (2) when information provided by the geometry of the arena has successfully guided navigation in the past, then subsequent navigation using landmarks is retarded relative to a group who initially navigated using landmarks. Analysis of path length data revealed that the longer latencies noted in the two ED groups, relative to the appropriate ID groups, did not reflect a general slowing. Instead, the longer latencies observed in the former groups were, at least in part, caused by increased distances traversed in the ED groups relative to the ID groups. Result 1 is difficult to reconcile with the proposals of Doeller and Burgess (2008), who suggested that learning about the boundary of the environment is impervious to the influence of learning about information from landmark information and, importantly, that learning relative to boundaries occurs independent of behavioral error. Results 1 and 2 are difficult to reconcile with both Cheng’s (1986) modular analysis of spatial learning and Miller and Shettleworth’s (2007, 2008) associative theory of spatial learning. The former theory proposes that geometric information is encoded in a module that can neither influence, nor be influenced by, learning about landmarks. The latter theory proposes that attention paid to navigational elements is fixed, thus precluding it from explaining any demonstration of a spatial ID-ED effect.

The stimuli employed as landmarks in Experiment 1 were colored panels that were spatially integrated into the boundaries of the arenas during Stages 1 and 2. This choice of stimuli has a number of theoretical implications, two of which we consider now. First, it has been suggested that learning may result in the acquisition of orienting responses to cues that are important to the solution of a discrimination (Spence, 1940, 1952). If these cues are subsequently established as unimportant to the solution of a discrimination (as in the case of an ED shift), then acquisition will be retarded because orienting responses will be made to the (now) irrelevant cue, potentially hindering the perception of the relevant cue. This analysis shifts the locus of the effect of the ID-ED shift to a more peripheral orienting mechanism than the analysis of the effect provided by theories of learning such as Mackintosh’s (1975) theory, which assumes the effect is the consequence of a more central change in the attention that is paid to a stimulus despite it being perceived. By demonstrating, here, an ID-ED effect when the features of the arena relevant to finding the goal are spatially integrated with the features of the arenas that are irrelevant makes it unlikely that that the current results were a consequence of a more peripheral strategy (cf. Pearce, Esber, George, & Haselgrove, 2008). Second, although colored wall panels have been considered as landmarks by some authors (e.g., Pearce et al., 2006), it seems entirely reasonable to argue that such features are integral components of the boundary of the arena (e.g., Wilson & Alexander, 2010). If this is accepted, then it may be argued that Experiment 1 only goes so far as to demonstrate that information contained within a geometric, or boundary, module is able to interact—a possibility that is not entirely ruled out by analyses such as those proposed by Cheng (1986) and Doeller and Burgess (2008). Experiment 2 was therefore conducted to address this matter and examined whether discrete landmarks that are spatially separated from the arena boundary can influence navigation that is based on information that is provided by its shape (and vice versa).

## Experiment 2

Experiment 2 replicated the design of Experiment 1, but in place of colored wall panels, colored spheres that were present in each corner of an arena served as landmarks. The spheres were spatially separated from the boundaries of the environment, such that in a horizontal plane the full 360° of the sphere could be viewed, and were suspended at a height that enabled participants to walk under them. In Stage 1, four spherical landmarks of different shades of blue were located in the four corners of the kite-shaped arena used in Experiment 1. In Stage 2, four spherical landmarks of different shades of red were located in the four corners of the trapezium-shaped arena used in Experiment 1. For group Shape–Shape, the hidden goal was again always located in the same corner of the kite, and the same corner of the trapezium, no matter which landmark was present in that corner in either arena. For group Landmark–Landmark, the goal was always under the same landmark in the kite or trapezium, no matter which corner the landmark occupied in each arena. For group Shape–Landmark, the hidden goal remained in the same corner of the kite no matter what landmark was present in the corner, but in the trapezium the goal then remained under the same landmark no matter which corner it was in. Finally, for group Landmark–Shape, the hidden goal remained under the same landmark in the kite shaped arena no matter which corner the landmark was in, but in the trapezium remained in one corner no matter which landmark was present in that corner.

### Method

#### Participants

Thirty-two participants were recruited from the University of Nottingham (24 females). Participants were again randomly allocated to one of the four groups in the experiment and were given course credit or £5 in return for participation. The age of participants ranged from 18 to 37 years (*M* = 21.2, *SEM* = 0.84). An additional £10 was awarded to the participant who completed the experiment in the shortest time.

#### Materials

The monitor, computer equipment, and all arenas were exactly the same as those used in Experiment 1, with the exception of the landmarks that, for the current experiment, were discrete spheres 90 cm in diameter instead of colored wall panels. The spherical landmarks were constructed using Blender software (www.blender.org) and were imported into Mazesuite. The blue spheres used in Stage 1 of the experiment were defined as RGB: 0.000, 0.540, 0.640; 0.159, 0.326, 0.800; 0.000, 0.123, 0.720; and 0.000, 0.464, 0.800; and the red spheres used in Stage 2 were defined as 0.635, 0.239, 0.640; 0.640, 0.000, 0.392; 0.512, 0.000, 0.314; and 0.238, 0.131, 0.465. Within the arenas, the landmarks were 1.475 m away from the apex of each corner, on a notional line that bisected the corner in half. The walls of both the kite shaped and trapezium shaped arenas were a uniform cream color throughout the experiment (see Experiment 1, Method). Figure 1c shows a screen shot of an example of the trapezium-shaped arena used in Experiment 2 and Experiment 3.

#### Procedure

The procedure for Experiment 2 was identical to Experiment 1.

### Results and Discussion

**Figure 4 fig4:**
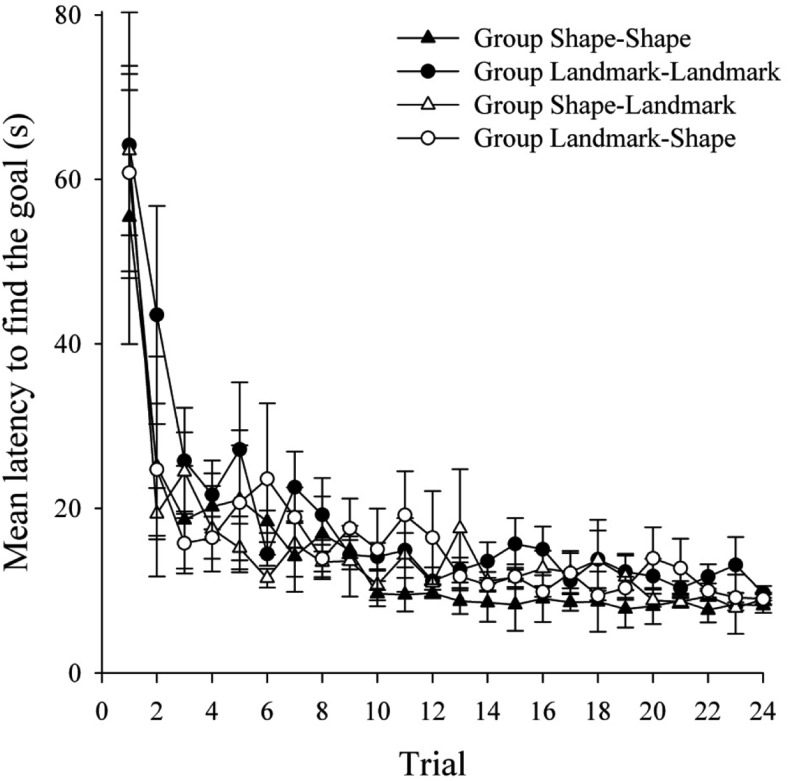
Mean latencies of the four groups to find the hidden goal in Stage 1 of Experiment 2. Error bars show 1 ± *SEM*.

Figure 4 shows the mean latency of the 4 groups to find the hidden goal during the 24 trials of Stage 1. In keeping with the results of Experiment 1, learning progressed at a similar rate in the four groups and the asymptotes of performance were similar. A two-way ANOVA of individual latencies to find the goal, with the variables of relevant cue in Stage 1 (landmarks or shape) and trial (1–24), revealed a significant main effect of trial, *F*(23, 690) = 26.11, *MSE* = 139.05. There was no main effect of relevant cue, *F*(1, 30) = 2.20, *MSE* = 727.87, and no significant interaction between relevant cue and trial, *F* < 1.[Fig-anchor fig4]

**Figure 5 fig5:**
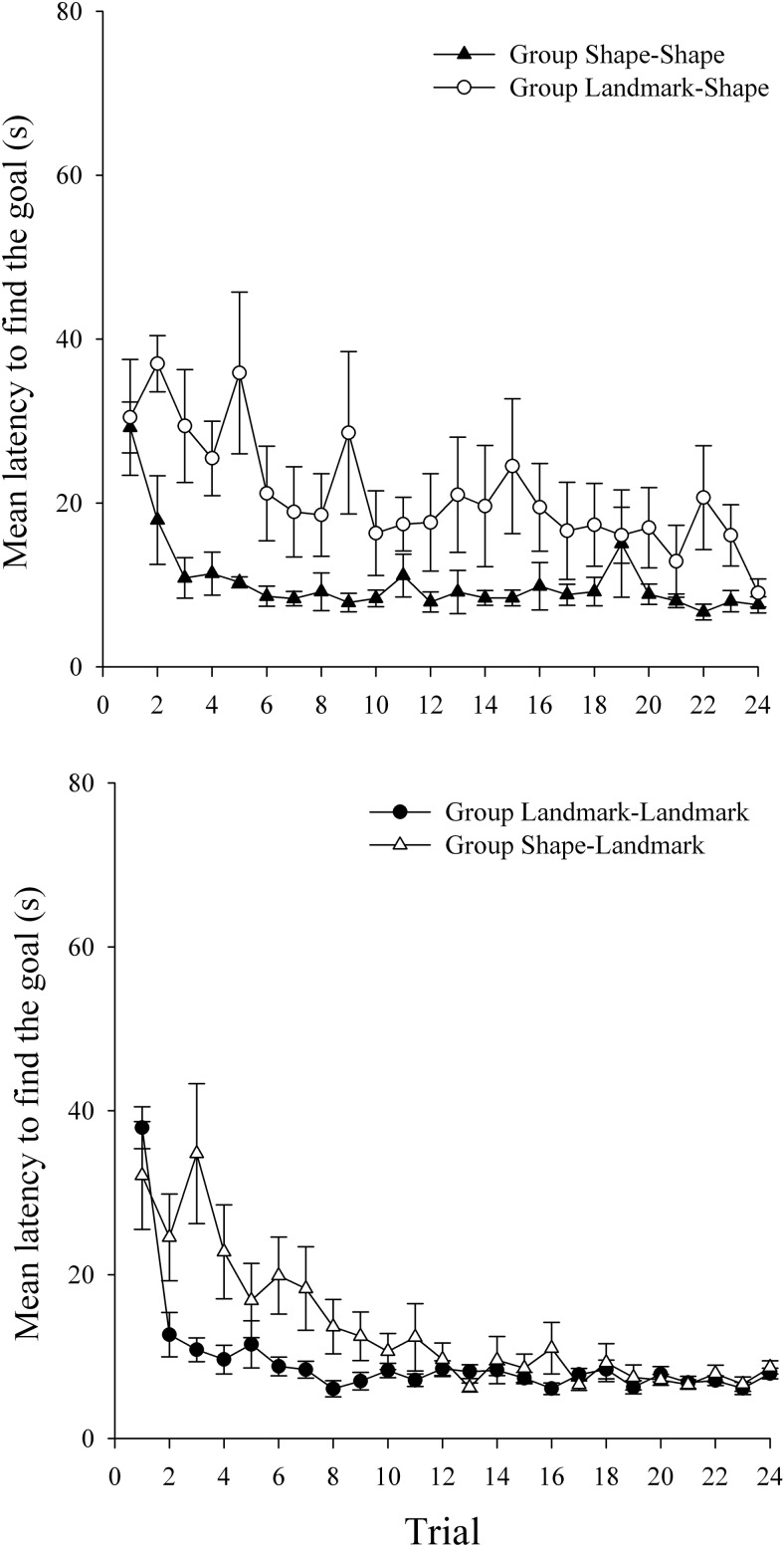
Top panel: mean latencies of groups Shape–Shape and Landmark–Shape to find the hidden goal in Stage 2 of Experiment 2. Bottom panel: mean latencies of groups Shape–Landmark and Landmark–Landmark to find the hidden goal in Stage 2 of Experiment 2. Error bars show 1 ± *SEM*.

The mean latencies to find the goal during Stage 2 are shown in the top panel of Figure 5 for groups Shape–Shape and Landmark–Shape, and in the bottom panel of Figure 5 for groups Landmark–Landmark and Shape–Landmark. In keeping with the results of Experiment 1, both groups that performed an ED shift (groups Landmark–Shape and Shape–Landmark) showed longer latencies to find the goal relative to the appropriate ID groups (groups Shape–Shape and Landmark–Landmark, respectively). There was an indication that this effect was more sustained in the groups undergoing shape relevance training in Stage 2 than groups who groups undergoing landmark relevance training in Stage 2. A three-way ANOVA of individual latencies to find the goal, with the variables of shift (ID or ED), relevant cue in Stage 2 (shape or landmarks), and trial (1–24), revealed a significant main effect of trial, *F*(23, 644) = 12.70, *MSE* = 76.72, and a significant main effect of shift, *F*(1, 28) = 10.92, *MSE* = 968.61, confirming that those performing an ED shift were, overall, slower to find the goal than those performing an ID shift. The main effect of relevant cue approached significance, *F*(1, 28) = 3.69, *p* = .065, which indicated that there was a trend toward participants finding the goal quicker when landmarks were relevant compared to when shape was relevant. Importantly, a significant Shift × Trial interaction was obtained, *F*(23, 644) = 3.13, *MSE* = 76.72. Simple main effects analysis revealed that, overall, participants performing an ED shift were significantly slower to find the goal than participants performing an ID shift on Trials 2–9, *F*s(1, 672) > 5.035, *MSE* = 11.89. The Relevant Cue × Shift interaction was not significant, *F*(1, 28) = 2.18, *MSE* = 968.61, and the Relevant Cue × Trial interaction was also not significant, *F*(23, 644) = 1.43, *p* = .08, although there was a trend for groups navigating on the basis of landmark to learn Stage 2 quicker than groups navigating on the basis of the shape of the arena. Finally, the three-way interaction was not significant, *F* < 1.[Fig-anchor fig5]

As with Experiment 1, full path length analyses for Experiment 2 can be viewed in the online supplemental materials to this article. We note here that individual distances traversed were treated with a three-way ANOVA, which incorporated variables of shift (ID or ED), relevant cue in Stage 2 (shape or landmarks), and trial (1–24). A significant interaction between shift and trial was again obtained, *F*(23, 644) = 2.70, *MSE* = 259.87. Participants in the ED groups traversed significantly greater distances to find the goal, compared to the ID groups, on Trials 2–7, 9, and 15, *F*s(1, 672) > 4.01, *MSE*s < 331.86.

The results of Experiment 2 replicate and extend the generality of the results from Experiment 1: Participants were slower to find a hidden goal when the cues relevant to navigation were from a dimension that had previously been irrelevant, rather than relevant, for navigation. Again, the longer latencies observed in the ED groups relative to the ID groups were, at least partly, due to the longer distance traversed in the former groups relative to the latter. Experiment 2 used intra-arena stimuli that were spatially separated from the arena boundary as landmarks, instead of the colored wall panels employed in Experiment 1. It is difficult to argue that these stimuli were encoded by participants as boundary information. It thus seems that the current experiment constitutes a demonstration that learning about a landmark interfered with learning about the geometric properties of an arena. These results are, therefore, inconsistent with theories that suggest boundary cues enjoy a special status, in that learning to them does not follow general associative principles of behavioral error and are not susceptible to interference from local landmark information (e.g., Doeller & Burgess, 2008), or theories that emphasize a similar special status for geometric information (e.g., Cheng, 1986; Gallistel, 1990).

The retardation of navigation observed in the two ED groups (groups Landmark–Shape and Shape–Landmark) was, of course, a retardation relative to navigation in the two ID groups (groups Shape–Shape and Landmark–Landmark). It is conceivable, therefore, that the results of Experiment 1 do not reflect a retardation of learning in the ED groups. In keeping with the proposals of modular theories of geometric navigation (e.g., Cheng, 1986), it is possible that navigation in the two ED groups in Stage 2 was, in fact, entirely un-affected by navigation in Stage 1. The difference observed between the ID and ED groups could, instead, reflect a facilitation of learning in the two ID groups—a possibility that is not explicitly prohibited by the aforementioned theories. This analysis encounters difficulty when explaining exactly why navigation should be facilitated in group Shape–Shape. The geometric features of the goal location in Stage 1 were deliberately chosen so as to not convey any advantage to participants when they moved to Stage 2 of the experiment. Thus, if the goal was in an acute (or obtuse) corner in Stage 1, then it was located in an obtuse (or acute) corner in Stage 2. Similarly, if the goal was located, for example, in a corner that had a short wall to the left of a long wall in Stage 1, then it was located in a corner that had a long wall to the left of a short wall in Stage 2. Thus, any direct transfer of geometric information pertaining to the goal location from Stage 1 to Stage 2 would, if anything, hinder, rather than facilitate navigation.

For Experiment 1, the landmarks were spatially integrated into the corners of the arena boundary, whereas in the current experiment, the landmarks were displaced from the arena boundaries. The results of Experiment 2 would therefore seem to be open to the peripheral orienting account described in the discussion of Experiment 1. Although it is not possible to fully rule out this analysis for Experiment 2, as can be seen in Figure 1c, the landmarks were located sufficiently close to the corners of the arena that any orienting response made toward a landmark cue coincided with an orienting response toward the geometry of the corner that the landmark occupies. Similarly, any orienting response made toward a given corner of the arena will coincide with an orienting response toward the landmark placed in that corner. On this basis, therefore, it seems unlikely that peripheral orienting mechanisms provide an adequate explanation of the pattern of results observed.

## Experiment 3

At face value, Experiment 2 seems to constitute a challenge to theories of navigation that confer a special status to the global shape of the environment. However, Experiment 2 failed to provide any evidence that, as a consequence of navigation, participants acquired a global representation of the shape of the arena—a representation that “cognitive map” theories (e.g., Cheng, 1986; O’Keefe & Nadel, 1978) predict will be extracted as a consequence of navigation. It is difficult to know, therefore, how much of a challenge Experiment 2 poses to these theories. This issue is particularly salient when one considers the results of spatial learning experiments conducted with rats, which provide evidence for a much more local encoding of geometry. Pearce, Good, Jones, and McGregor (2004), for example, trained rats to find a hidden goal in the corner of a rectangular arena in which the right hand wall was long and the left hand wall was short. Pearce et al. suggested that if subjects had used the global features of the rectangle to find the hidden goal, then placing them in a novel kite-shaped arena should disrupt performance. The results of test trials in the kite-shaped arena did not conform to this prediction: Rats preferentially searched first in the corner of the kite in which the right hand wall was long and the left hand wall short (see also Cheng, 2005; McGregor, Jones, Good, & Pearce, 2006). Pearce et al. suggested that the local geometric features that were common to both the rectangular and kite-shaped areas (e.g., the conjunction of a short wall and a long wall) were used to guide navigation. However, these results do not rule out the possibility that, in addition to the encoding of local features, global boundary information (unique to the rectangular arena) was also encoded during training. By the same token therefore, any experiment that is claimed to constitute a challenge to the assumptions of theories of navigation which assume the presence of a global representation should also comprise some evidence for such a global representation—evidence that Experiments 1 and 2 (as well as other related cue competition experiments; e.g., Pearce et al., 2006) are lacking. Experiments 3a and 3b sought to replicate the findings of Experiment 2, in addition to assessing whether participants formed any knowledge of the global shape of the arena and, more importantly, whether this information was influenced by the relevance training provided by the ID-ED task. Experiment 3a was an exact replication of Experiment 2, but with the addition, at the end of the experiment, of a shape recognition task following Stage 2 of the ID-ED task. Experiment 3b was a close replication of Experiment 2, except that the participants began by navigating in the trapezium shaped arena in Stage 1, following which participants navigated in the kite shaped arena during Stage 2. The shape recognition task was also administered at the end of Experiment 3b. For the shape recognition task at the end of Experiments 3a and 3b, participants were presented with black and white “target” pictures of a kite and a trapezium and “distracter” stimuli, similar in form to the targets (a triangle and a parallelogram, respectively). Participants were required to report whether or not the shapes presented at test matched those explored during the preceding navigation stages. If the training in Stages 1 and 2 of this experiment permitted participants to extract a global representation of the shapes of the kite and trapezium shaped arenas, then they should be able to distinguish these targets from the distracters. This being the case, it would provide evidence for the presence of global encoding of the shape of the arena as a consequence of exploration within it. At the same time, however, if performance on this recognition task were influenced by relevance training with landmarks then we would also have evidence that a global representation of shape is susceptible to interference from local landmarks—a possibility that, as we have outlined earlier, is prohibited by a variety of theories of spatial navigation (e.g., Cheng, 1986; Doeller & Burgess, 2008; Gallistel, 1990).

### Method

All procedural, material, and apparatus details for the navigation stages of Experiment 3a were identical to those reported in Experiment 2. Experiment 3b was also identical to Experiment 2, except that the order of arenas was reversed, thus, counterbalancing the order of presentation of arenas. For clarity, during Stage 1 of Experiment 3b, participants completed 24 trials in the trapezium shaped arena which contained four red landmarks, and, in Stage 2, participants completed 24 trials in the kite shaped arena which contained four blue landmarks. Only details pertaining to the shape recognition task are reported in the following section.

#### Participants

A total of 96 participants were recruited from the University of Nottingham (44 males). Participants were again given course credit or £5 in return for participation. An additional £10 was awarded to the participant who completed each experiment in the shortest time.

##### Experiment 3a

The age of participants ranged from 18 to 47 years (*M* = 22.79, *SEM* = 0.71). Participants were allocated to each of the four groups in pseudo-random manner to ensure that an equal number of males (6) and females (6) were present in each group.

##### Experiment 3b

The age of participants ranged from 18 to 30 years (*M* = 20.52, *SEM* = 0.34). Participants were again pseudo-randomly allocated to each group to ensure that the same number of males (5) and females (7) were in each group.

#### Materials

Black-lined and white-filled pictures of a kite (on screen wall lengths of 35 mm and 108 mm) and trapezium (on screen wall lengths of 35 mm, 108 mm, and 69 mm) were created using Microsoft PowerPoint 2007. Pictures of an isosceles triangle (on screen wall lengths of 108 mm and 60 mm) and a regular parallelogram (on screen wall lengths of 86 mm and 60 mm) were also created as distracter stimuli. All stimuli were presented on a white background. This task was run on a standard sized (476.6 mm × 268.1 mm) computer monitor. Experimental events were controlled and responses recorded by Psychopy (Peirce, 2007; www.psychopy.org).

#### Procedure

Following the ID-ED task, the shape recognition task was administered, during which participants were then sat not more than 1 m in front of a standard sized computer monitor and presented with the following, on screen instructions:

For the final stage of the experiment you will be presented with pictures of different shapes. It is your task to decide which of these shapes match the shapes of the arenas that you previously navigated.

Please press “Y” if you think you were in the shape before.

Please press “N” if not.

Take as much time as you need to make your decision.

<Press the space bar to continue>

On each trial a kite, trapezium, triangle, or parallelogram was presented in the center of the computer monitor. Each picture was presented in two different orientations during the task. The kite and triangle were each presented once with their most acute corner facing the left hand side of the computer monitor, and once with their most acute corner facing the right hand side of the computer monitor. The trapezium was presented once with its smallest side facing the top of the monitor and once with its smallest side facing the bottom of the monitor. On both trials, the parallelogram was presented with its two longest sides running parallel to the top of the monitor. On one trial, the two acute corners were to the top right and bottom left of the shape, on the other trial the two acute corners were to the top left and bottom right of the shape. The order of presentation of the eight stimuli was randomized independently for each participant. Below each picture, centered, were the following on screen instructions:

Were you in this shape? (Y/N)

Trials were self-paced, with each trial terminating when the participant pressed either the “Y” or “N” key. The subsequent trial began immediately after the termination of the preceding trial.

After all eight trials, the screen was cleared, and participants received on screen instructions to contact the experimenter.

### Results and Discussion

As Experiments 3a and 3b were two halves of a counterbalanced procedure, data from the two experiments were collapsed together in both the analysis of navigational behavior, and in the analysis of the shape recognition task.

#### Intradimensional–extradimensional shift

**Figure 6 fig6:**
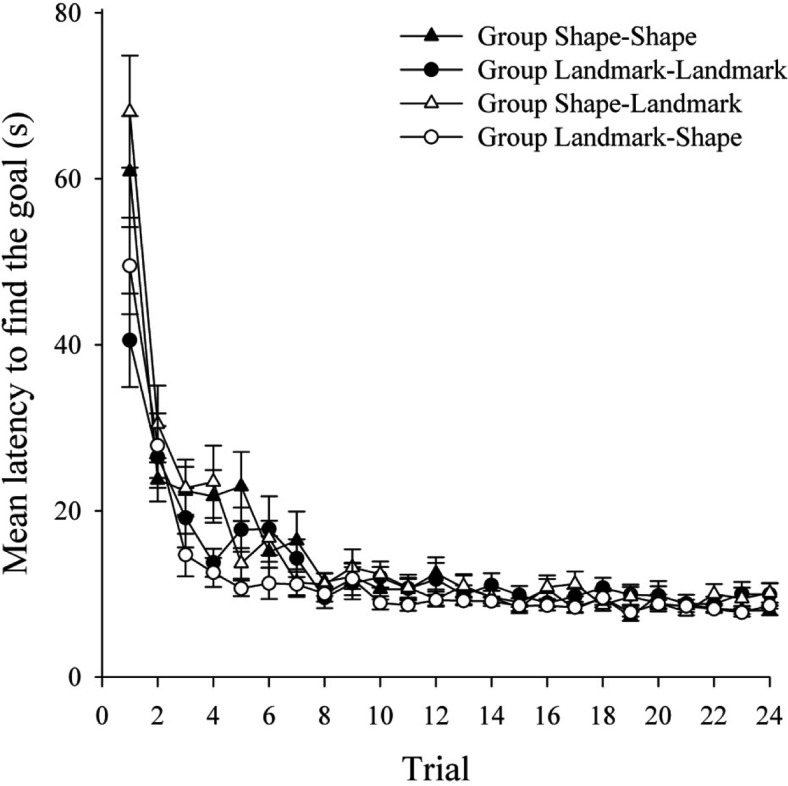
Mean Latencies of the four groups to find the hidden goal in Stage 1 of Experiment 3. Error bars show 1 ± *SEM*.

Figure 6 shows the latency to find the hidden goal, in seconds, during the 24 trials of Stage 1 in the four groups. All groups showed a reduction in the latency to find the goal as trials progressed, although groups for which landmarks were relevant found the goal quicker early in training. A two-way ANOVA of individual latencies to find the goal, with variables of relevant cue in Stage 1 (landmarks or shape) and trial (1–24), revealed significant main effects of relevant cue, *F*(1, 94) = 4.37, *MSE* = 521.67; trial, *F*(23, 2162) = 96.82, *MSE* = 95.33; and a significant interaction between relevant cue and trial, *F*(23, 2162) = 4.80, *MSE* = 95.33. Simple main effects analysis of the interaction revealed that groups for which landmarks were relevant were quicker to find the goal on Trials 1 and 4 only, *F*s(1, 94) > 9.75, *MSE*s < 931.28.[Fig-anchor fig6]

**Figure 7 fig7:**
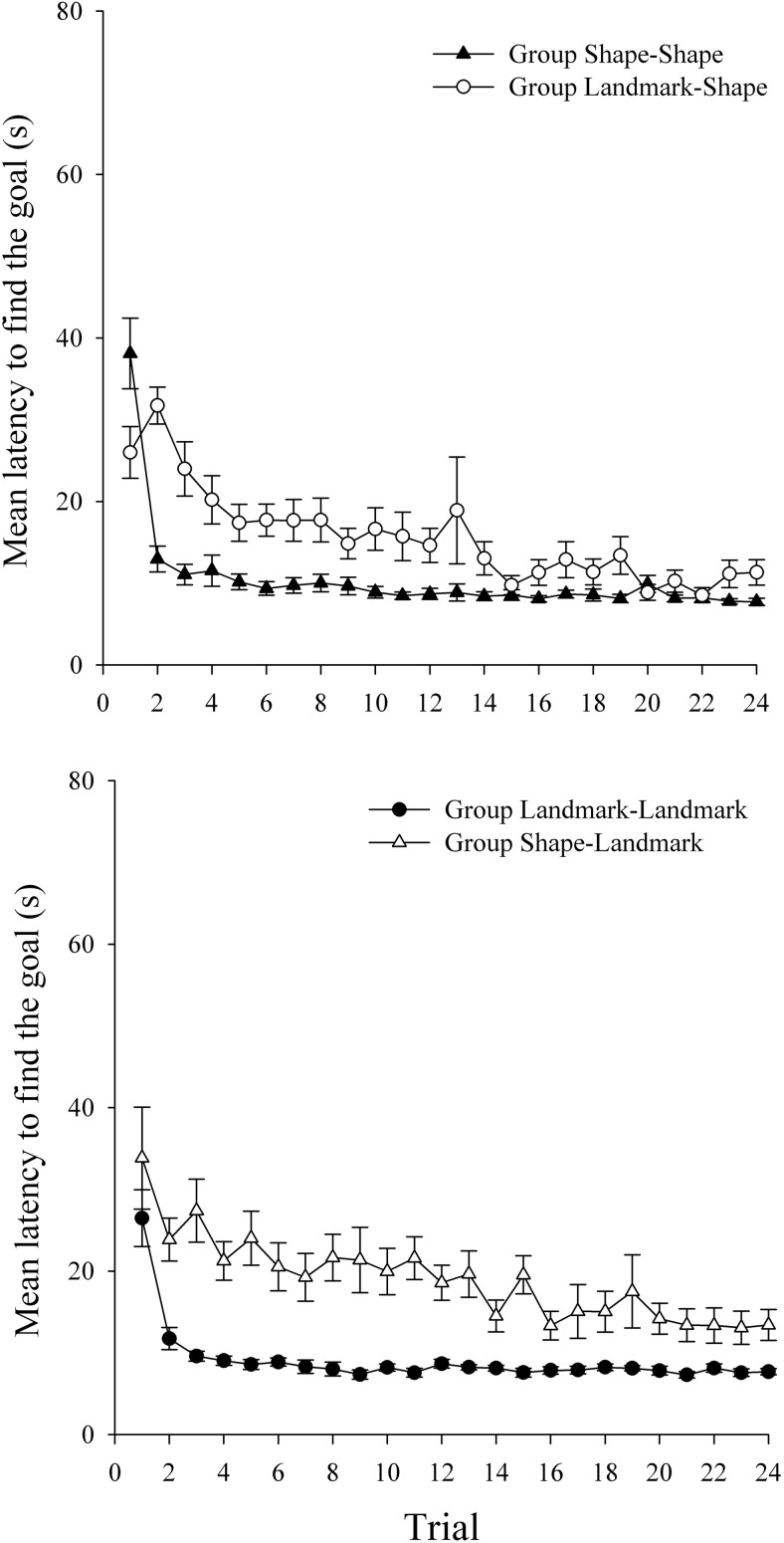
Top panel: mean latencies of groups Shape–Shape and Landmark–Shape to find the hidden goal in Stage 2 of Experiment 3. Bottom panel: mean latencies of groups Shape–Landmark and Landmark–Landmark to find the hidden goal in Stage 2 of Experiment 3. Error bars show 1 ± *SEM*.

The mean latencies to find the goal during Stage 2 are shown in the top panel of Figure 7 for groups Shape–Shape and Landmark–Shape, and in the bottom panel of Figure 7 for groups Landmark–Landmark and Shape–Landmark. In keeping with the results of Experiments 1 and 2, both groups that performed an ED shift (groups Landmark–Shape and Shape–Landmark) showed longer latencies to find the goal relative to the appropriate ID groups (groups Shape–Shape and Landmark–Landmark, respectively). A three-way ANOVA of individual latencies to find the goal, with the variables of shift (ID or ED), relevant cue in Stage 2 (shape or landmarks), and trial (1–24), revealed significant main effects of shift, *F*(1, 92) = 57.00, *MSE* = 580.17; trial, *F*(23, 2116) = 23.61, *MSE* = 85.62; but not relevant cue, *F* < 1. Importantly, the interaction between shift and trial was significant, *F*(23, 2116) = 4.83, *MSE* = 85.62. Simple main effects analysis of this interaction revealed that while there was no difference between the ID and ED groups on Trial 1, *F* < 1, the ED groups were significantly slower to find the goal on Trials 2–24, *F*s(1, 92) > 4.81, *MSE*s < 311.26. The two-way interaction between relevant cue and trial was not significant, *F* < 1, but the interaction between relevant cue and shift was significant, *F*(1, 92) = 5.50, *MSE* = 580.17. Simple main effects analyses revealed that, for both landmark and shape relevance, the ED groups were significantly slower to find the goal in Stage 2, overall, than the ID groups, *F*s(1, 92) > 13.55, *MSE*s = 24.17. There was no difference in the time taken to find the goal during Stage 2, overall, in the ID groups, *F* < 1, although in the ED groups, the Landmark–Shape group was, overall, quicker to find the goal in Stage 2 compared to the Shape–Landmark group, *F*(1, 92) = 5.54, *MSE* = 24.17. Finally, the three-way interaction between shift, relevant cue, and trial was not significant, *F*(23, 2116) = 1.45, *MSE* = 85.617.[Fig-anchor fig7]

Full path length analyses can again be viewed in the online supplemental materials to this article. The three-way ANOVA of individual distances traversed to find the goal, with the variables of shift (ID or ED), relevant cue in Stage 2 (shape or landmarks), and trial (1–24), yielded a significant interaction between shift and trial, *F*(23, 2116) = 4.32, *MSE* = 351.13. Simple main effects analysis revealed that, on Trial 1, there were no differences in the distances traversed by the ID and ED groups, *F* < 1, but that the ED groups traversed a significantly greater distance to find the goal than ID groups on Trials 2–19 and Trials 21–24, *F*s(1, 92) > 6.03, *MSE*s < 1,398.40.

#### Recognition task

During the recognition test, it is possible that the two distractor stimuli (parallelogram and triangle) both acted as foils for each of the two target stimuli (kite and trapezium). As such, the total number of “Yes” responses to the kite target pictures and “No” responses to the triangle and parallelogram distracter pictures were summed and were divided by the total number of responses made to these pictures to calculate a percent correct score for the kite arena. Similarly, the total number of “Yes” responses to the trapezium target pictures and “No” responses to the triangle and parallelogram distracter pictures were summed, and dividing this number across the total number of responses made to these pictures to calculate a percent correct score for the trapezium arena.

**Figure 8 fig8:**
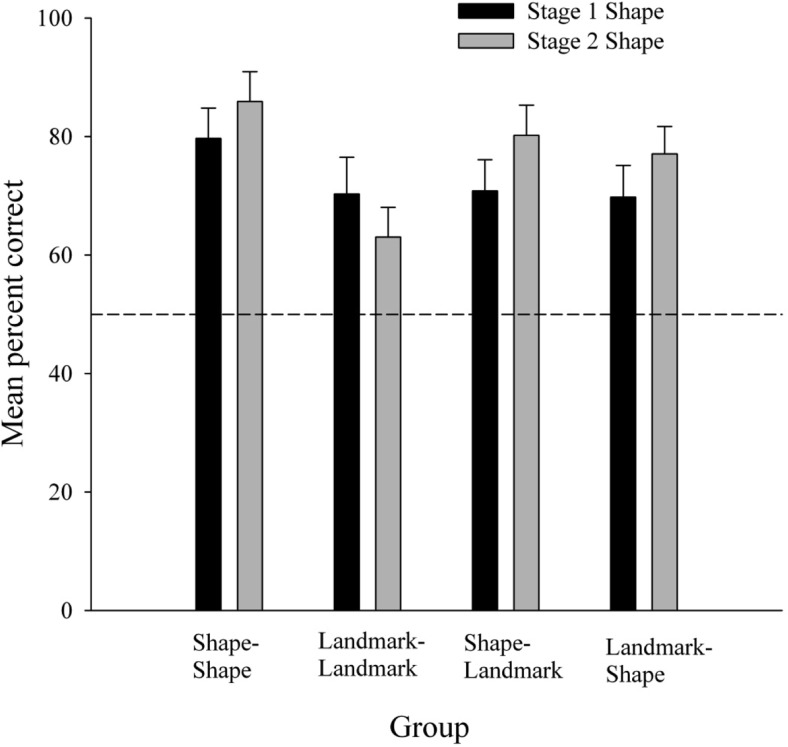
Mean percent correct recognition of the shapes navigated during Stage 1 and Stage 2 in the four groups of Experiment 3. Error bars show 1 ± *SEM*.

Figure 8 shows the mean percent correct recognition for the shapes navigated in Stage 1 and Stage 2 for each of the four groups. First, and consistent with the notion that navigation permitted the extraction of global representations of the shapes of the arenas, recognition of the Stage 1 and Stage 2 target shapes was good in all four groups. It appeared though, that while both ED groups displayed equivalent performance, group Shape–Shape had higher recognition scores than group Landmark–Landmark. First, one sample *t*-tests were conducted to assess if individual recognition scores for the navigated shape in Stage 1 and Stage 2 of the experiment were above chance. In the shape recognition task, four out of the eight presented shapes matched the navigated arenas, giving a chance level of 50%. However, in the calculations previously described, a maximum of two correct “Yes” responses to target shapes were summed with four responses made to the distracter pictures, giving a chance level of 33.33%. Taking the conservative value of a 50% chance level, recognition of the navigated shapes in both Stage 1 and Stage 2 were above chance in all four groups, *t*s(23) > 3.33. Second, individual percent correct scores were treated with a three-way ANOVA, with variables of shift (ID or ED), relevant cue in Stage 1 (shape or landmarks), and arena (Stage 1 or Stage 2). This revealed no significant effects of shift or arena, *F*s(1, 92) < 1.20, *MSEs* < 756.41, although there was an effect of relevant cue, *F*(1, 92) = 4.98, *MSE* = 756.41. There was, however, a significant interaction between shift and relevant cue, *F*(1, 92) = 4.98, *MSE* = 756.41. Simple main effects analysis of this interaction revealed a significant difference between the Shape–Shape and Landmark–Landmark groups, *F*(1, 92) = 9.95, *MSE* = 378.20; participants in the Shape–Shape group displayed significantly better recognition of the navigated shapes compared to participants in the Landmark–Landmark group. There were no differences in shape recognition between the Shape–Shape and Landmark–Shape group, the Landmark–Landmark and Shape–Landmark group, or the Shape–Landmark and Landmark–Shape groups, *F*s(1, 92) < 2.57, *MSE*s = 378.20. Returning to the results of the overall ANOVA, the Shift × Arena and Relevant Cue × Arena interactions were not significant, *F*s(1, 92) < 1.54, *MSE*s = 271.80, and the three-way interaction was also not significant, *F* < 1.[Fig-anchor fig8]

In keeping with the results of Experiment 2, navigating on the basis of stimuli drawn from one dimension retarded subsequent navigation if the relevant stimuli were drawn from a different dimension, in terms of both time taken and distance traversed to find the hidden goal. To reiterate a point made earlier, the retardation of group Landmark–Shape relative to group Shape–Shape is not predicted by theories that state boundary information is processed in a fashion immune to interference from learning about landmarks (e.g., Cheng, 1986; Doeller & Burgess, 2008; Gallistel, 1990) or by the theory proposed by Miller and Shettleworth (2007, 2008).

Experiment 3 is particularly novel in its use of the final shape recognition test to assess participant’s global representation of the shapes of the arenas navigated. Importantly, and contrary to theories that suggest this knowledge is acquired independently of the presence of the other cues, knowledge of the global structure of the shape of the environments was modulated by varying the relevance of the shape and/or the landmarks. The Shape–Shape group displayed good recognition of the target stimuli following training in which the shape of the arena was relevant to finding the goal throughout the experiment. Training in which the shape of the arena was irrelevant for finding the goal throughout the experiment limited the extent to which the global structure of the boundaries was encoded and, ultimately, rendered it less recognizable at test for the Landmark–Landmark group. Clearly then, acquisition of knowledge about the global boundary structure of an environment is affected by the presence of other, non-boundary, cues. It is, perhaps, not surprising that the recognition scores in the ED groups did not differ considering that, in both groups, for one half of the experiment, the boundary shapes of the arena were relevant to finding the goal, whereas for the remainder of the experiment the landmarks were relevant.

## General Discussion

In three experiments, participants were required to find a hidden goal in a virtual arena that contained distinctive landmarks. Either the shape of the arena, or the location of the landmarks, was made relevant to navigating toward the hidden goal. In each experiment, participants were faster to find the goal, and traversed a shorter distance to find the goal, when the dimension relevant to finding the goal was the same as during previous sessions of navigation. These results were obtained when the landmarks were spatially integrated into the boundary of the arena (Experiment 1), or when they were spatially separated from the boundary as intra-arena cues (Experiments 2 and 3). Experiment 3 revealed that participants’ ability to recognize the shape of the arenas that they had previously navigated was influenced by whether shape had been established as relevant to finding the goal during the experiment.

As we have noted earlier, these results are difficult to reconcile with theories of spatial learning that place an emphasis on the special status of the shape of an arena in navigation. According to a number of theories (e.g., Cheng, 1986; Gallistel, 1990), learning about the shape of an arena involves the acquisition of a representation of the geometric relations of the arena that is impervious to interference from learning about landmark information. The results of Experiment 3 are, in particular, relevant to this suggestion. Participant’s recognition of the overall shape of the arenas was significantly greater than chance, a result compatible with the formation of a global representation of the geometry of the arenas. However, recognition of the navigated arenas in the experiment was impaired if landmarks were relevant throughout the duration of the experiment, relative to if shape was relevant throughout the experiment. Previous studies of the interaction of landmarks and shape cues in studies of either human or animal spatial learning have not reported any measure of participants’ knowledge of the shape of the arena previously navigated (e.g., Doeller & Burgess, 2008; Pearce et al., 2004; Redhead & Hamilton, 2009). To the best of our knowledge, therefore, the current results constitute the first demonstration of an interference of the global representation of the shape of an arena by local landmarks.

The results of the current experiments permit further constraints to be placed upon explanations of spatial navigation that have, as their basis, associative theories of learning. The ID-ED effects noted in the three experiments here are inconsistent with the proposals of Miller and Shettleworth (2007, 2008). Their model assumes that the salience of stimuli (α) is fixed, and for an associative model to be capable of explaining ID-ED effects, changes in the attention paid to relevant and/or irrelevant dimensions must be permitted (Le Pelley, 2004; Mackintosh, 1975). Mackintosh’s (1975) theory of associative learning is an example of an associative theory that does exactly that. According to Mackintosh, the change in the associative strength of a target cue (ΔV_T_) progresses according to Equation 3, which is similar to Equation 1:

$$\Delta {\text{V}}_{\text{T}}=\;\alpha_{\text{T}}\left(\lambda -{\text{V}}_{\text{T}}\right)$$ 3

Here, α_T_ is the attention paid of the target cue, β is a learning rate parameter determined by the properties of the outcome, and λ is the asymptote of learning supported by the outcome. Crucially, according to Mackintosh (1975), the attention paid (α) to a cue increases if it is a better predictor of the outcome than all other cues present on a trial and decreases if it is no better a predictor of the outcome than all the other cues present on a trial. The rules specified by Mackintosh for determining these increases and decreases to a target cue (T) are shown in Equations 4a and 4b:

$$\Delta \alpha_{\text{T}}>\;0\;\text{if}\;\left|\lambda -{\text{V}}_{\text{T}}\right|\;<\;\left|\lambda -{\text{V}}_{\text{r}}\right|\;$$ 4a

$$\Delta \alpha_{\text{T}}<0\;\text{if}\;\left|\lambda -{\text{V}}_{\text{T}}\right|\;\geq \;\left|\lambda -{\text{V}}_{\text{r}}\right|\;$$ 4b

where V_r_ is the sum of the associative strength of all available cues, minus V_T_ (that is to say, the remainder). The size of the change in α is assumed to be proportional to the magnitude of the inequalities in Equations 4a and 4b. Thus, cues which are good predictors of subsequent events will enjoy an increase in their salience—or attention. Irrelevant cues that are poor predictors of subsequent events, however, suffer a reduction in their attention (see also Esber & Haselgrove, 2011; Le Pelley, 2004). In order to explain instances of the ID-ED effect, Mackintosh (1975) proposed that attention generalizes among stimuli in proportion to their similarity (p. 292). Consequently, attention should generalize more between cues that are drawn from the same dimension (such as the common features of two different environmental shapes, or two different sets of landmarks) than between cues that are drawn from different dimensions. On the basis of these two proposals, it is relatively straightforward to understand why learning, in Stage 2, was slower in the two ED groups than the two ID groups. Training in Stage 1 in groups Landmark–Landmark and Landmark–Shape should ensure that, by the end of this stage, attention will be higher to the relevant landmarks within the arena than its irrelevant shape. In contrast, Stage 1 training in groups Shape–Shape and Shape–Landmark should ensure that attention is higher to the relevant shape of the arena than the landmarks within it. This training should benefit Stage 2 learning in groups Landmark–Landmark and Shape–Shape, as the high attention paid to the relevant cues in Stage 1 of the training will generalize to the cues that continue to be relevant in Stage 2. However, the same will not be true for groups Landmark–Shape and Shape–Landmark. For these two groups, the high attention acquired to the relevant cues in Stage 1 will generalize to cues that are subsequently irrelevant to learning in Stage 2—hindering performance in the task.

In addition to providing an explanation for the ID-ED effects observed in the experiments reported here, Mackintosh’s (1975) model might also be able to provide a reconciliation of the conflicting findings from spatial overshadowing experiments that were presented in the introduction. According to Mackintosh’s theory, cues which enter the experiment with, inherently, high salience will enjoy gains in attention if they are learned about in compound with a cue that is of a lower inherent salience (which itself will suffer a loss in attention). This process will permit the cue that is more salient to overshadow the less salient cue, but not vice versa. It is possible, therefore, that failures of a landmark to overshadow a boundary shape may be due to the landmark possessing low unconditional salience relative to the shape and, likewise, successes of landmarks overshadowing boundary shape may be due to the landmark possessing high unconditional salience relevance to the shape. Such one-way overshadowing is not uncommon in the non-spatial literature (e.g., Mackintosh, 1975; Miles & Jenkins, 1973), and recent work within the spatial field has linked the relative salience of landmarks and shape cues to the direction of overshadowing that is observed (see Kosaki, Austen, & McGregor, 2013; Redhead, Hamilton, Parker, Chan, & Allison, 2012). Such failures of overshadowing are not only limited to instances of salience asymmetry, however. If both cues enter the experiment with particularly high unconditional salience, the theory proposed by Mackintosh anticipates no overshadowing at all. Thus, if both the landmark and shape cues in previous overshadowing experiments were both of an unconditionally high salience, than the landmark would fail to overshadow learning based upon the shape of the boundary, and vice versa. Although evidence consistent with this prediction has been obtained in non-spatial domains (Mackintosh, 1976), it remains to be determined whether a comparable effect can be observed in spatial overshadowing experiments.

Our discussion thus far has focused on theories of navigation that have applied the principles of associative learning to the spatial domain (e.g., Miller & Shettleworth, 2007, 2008). However, it is appropriate to also consider the role of more explicit, verbally mediated, processing mechanisms in adult human spatial navigation. For example, it seems conceivable that participants in groups who received training in Stage 1 in which the shape of the kite was relevant to finding the goal (groups Shape–Shape and Shape–Landmark) may have acquired a declarative statement in the first stage of the experiment that assisted navigation. For example, “the goal is located in the corner of the arena where the long wall is to the left of the short wall, irrespective of the colour of the landmark that is there—so ignore that.” Acquisition of such a statement could be expected to (a) facilitate subsequent learning that is based upon the shape of a new environment and/or impede subsequent learning that is based upon landmarks (Experiments 1–3) and (b) mediate recognition performance on the basis of the “long wall is to the left of a short wall” feature that verbal representations of the training and test stimuli will have in common (Experiment 3). An experiment conducted by Hermer-Vazquez, Spelke, and Katsnelson (1999) seems to provide support for the role of explicit verbal mechanisms in spatial navigation. They required adult participants to locate a hidden goal in a rectangular room that had a blue panel attached to one of the shorter walls. Performance on this task was significantly attenuated when it was performed along with a verbal shadowing task but not a nonverbal rhythm-clapping task. However, an attempt to replicate this effect by Hupbach, Hardt, Nadel, and Bohot (2007) was not successful. Similarly, Ratliff and Newcome (2008) were unable to replicate Hermer-Vazquez et al. when the experiment was (a) appropriately counterbalanced and (b) preceded by clear instructions and a practice trial. Perhaps most problematic for advocates of the role of verbal mechanisms in spatial navigation is the observation by Bek, Blades, Siegal, and Varley (2010) that performance on the task described by Hermer-Vazquez et al. is comparable in participants with and without aphasia, even under conditions of verbal load where, presumably, any residual verbal competency is blocked. On the basis of these data, therefore, the contribution of explicit verbal encoding in spatial navigation is not compelling.

Alternatively, it is possible that controlled processing influences the impact of attentional processes on stimuli that are relevant or irrelevant to the solution of a task (e.g., De Houwer, Hermans, & Eelen, 1998; Posner & Snyder, 1975). A recent study by Le Pelley, Vadillo, and Luque (2013) is pertinent to this issue. In Stage 1 of their experiment, Le Pelley et al. employed a learned predictiveness task in which participants were required to categorize a compound of two stimuli into one of two different groups. The stimuli were drawn from two different dimensions (color or line orientation) with one of the dimensions of the compound being predictive of category membership, while the other dimension was irrelevant—a design formally equivalent to the training given to participants in Stage 1 of the current experiments. Once Stage 1 of their experiment task was complete, Le Pelley et al. employed a dot-probe task in which participants had to respond as quickly as possible upon presentation of the probe. Response times were faster to the probe when it had been spatially cued by a stimulus that was relevant, rather than irrelevant, to the categorization task in Stage 1—a result consistent with the idea that attention came to be biased more toward the relevant than the irrelevant stimulus. However, this effect was only observed when the interval between the stimuli from the categorization task and the probe was 250 ms. When this interval was 1,000 ms, the effect was abolished. Le Pelley et al. suggested that this finding was not consistent with the idea that learned changes in attention result in changes in controlled, effortful processing—otherwise the effect should be more, not less, pronounced with a longer inter-stimulus interval. Instead, Le Pelley et al. suggested that their results are more consistent with a rapid, associative process (e.g., Mackintosh, 1975). A comparable effect has also been demonstrated in the automatic evaluation of relevant and irrelevant information in adult human contingency learning (Le Pelley, Calvini, & Spears, 2013). It remains to be determined whether the results of the current experiments are a consequence of effortful, perhaps verbally mediated, cognitive processes, or instead more automatic mechanisms; indeed, it was not the goal of the current experiments to test between these two alternatives. However, converging evidence from studies that have looked either at the role of verbal processes in spatial navigation, or effortful versus automatic processing in learned predictiveness and irrelevance, provide little reason to expect the contribution of effortful, or verbally mediated, cognitive processes in the current experiments.

It is appropriate to consider, at this juncture, the relevance of the current experiments to other studies that have investigated the effects of stimulus relevance on learning, both in general, and more specifically in the domain of spatial learning. The results of many studies are now converging upon the conclusion that establishing a set of cues as relevant to acquiring a goal, or trial outcome, results in these cues acquiring more attention than the cues from another set that are irrelevant to acquiring the goal (for a review, see Le Pelley, 2010). As we have seen in the current experiments, as well as other demonstrations of the ID-ED effect (e.g., George & Pearce, 1999; Mackintosh & Little, 1969; A. C. Roberts, Robbins, & Everitt, 1988), learning about cues is faster when they have, in the past, been established as relevant rather than irrelevant predictors of goals—a result that is consistent with the idea that these cues are attracting more attention and are, hence, more associable (see also Le Pelley & McLaren, 2003). Furthermore, experiments have shown that relevant cues are less prone to the attentional blink than are irrelevant cues (Livesey, Harris, & Harris, 2009); they also support a superior “Posner cueing effect” (Le Pelley, 2010) and attract more eye gazes (Le Pelley, Beesley, & Griffiths, 2011) than irrelevant cues. Although studies of the influence of relevance training on stimulus attention are widespread in the non-spatial literature, we are aware of only two reports in which this issue has been studied in the domain of spatial learning, both of which investigated whether the associability of a cue is influenced by prior relevance training. The first report, by Trobalon et al. (2003), was outlined in the introduction. The second report, by Cuell, Good, Dopson, Pearce, and Horne (2012), trained rats in a Place group to find the location of a hidden goal with reference to the shape, and the extra-maze cues, of a distinctively shaped water maze, while laminated cards attached to the wall of the water maze were irrelevant. Rats in a Landmark group were required to find the goal with reference to laminated cards that were attached to the walls of the water maze, while the distinctive shape of the arena and extra-maze cues were irrelevant. During a subsequent test stage, place cues were relevant for a new discrimination. The results indicated that the place cues better controlled searching for the goal in the Place group than in the Landmark group. The results of the experiments presented here join this more general class of studies demonstrating the role of stimulus relevance on associability in spatial learning. Where they distinguish themselves, of course, is with the more specific conclusions that can be drawn about the influence of relevance training on the representation of the shape of the arena being navigated. Given that relevant cues have been shown to attract more eye gazes than irrelevant cues in studies of predictive learning in humans (Le Pelley et al., 2011), it would be interesting to assess if shape or landmark relevance training alters overt attention to these cue dimensions. Eye-tracking procedures, in which sampling times and distributions of visual foci are recorded, have been utilized in virtual navigation procedures previously (e.g., Hamilton, Johnson, Redhead, & Verney, 2009; Mueller, Jackson, & Skelton, 2008) and would offer a potential approach to address this issue.

Although modular theories of geometric information processing continue to be a matter of theoretical influence (e.g., Gallistel & Matzel, 2013; Jeffery, 2010; Spelke & Lee, 2012), it is relevant to note that Cheng has recently explored how a view-based navigational theory might succeed in explaining spatial navigation (Stürzl, Cheung, Cheng, & Zeil, 2008; see also Cheng, 2005, 2008; Cheng & Newcombe, 2005). The details of this analysis are beyond the scope of this article; however, in brief, this theory uses a function to determine the difference between the current global image and stored global images of nearby locations. Gradient descent is then used to model the movement of the organism away from the current position and toward locations successively closer to the goal. Although this theory has enjoyed some success in explaining how learning in an environment of one shape can transfer to an environment of another shape (Cheung, Stürzl, Zeil, & Cheng, 2008), the results of the current experiments may prove to challenge it, as the theory uses veridical images to represent the environmental stimuli, unadjusted for variations in attention. The theory proposed by Stürzl et al. (2008), therefore, seems to encounter the same problem when attempting to explain the basic ID-ED effect as Miller and Shettleworth’s (2007, 2008) model.

One problem that any theory of spatial navigation, associative or otherwise, has to address is how participants are able to correctly identify, from a novel perspective, the arena that had previously been navigated. Similar view-independent recognition effects have been reported elsewhere (e.g., Christou & Bulthoff, 1999; Hock & Schmelzkopf, 1980), but it must be acknowledged that, in the field of object recognition at least, demonstrations of complete viewpoint invariance are difficult to obtain (Farah, Rochlin, & Klein, 1994; Rock, Wheeler, & Tudor, 1989). Biederman (1987) suggested that an object (and by generalization, a view) could be recognized from a different perspective so long as the similarity between the views is sufficiently high, and so long as the relationship between the components of the views were not altered. Although the similarity of the components used during the navigation and recognition tests of Experiment 3 was particularly low, it is conceivable that recognition was achieved by matching the relationships between the components of the scenes. For example, during navigation within the kite-shaped arena, participants will encounter particular structural conjunctions of wall lengths (long–short, short–short, short–long and long–long)—the same conjunctions that are present in the plan view of this arena. Although it remains to be determined exactly how such conjunctions could be matched when the components upon which they are based are so different, the encoding of such structural information has been investigated and modeled from the perspective of associative learning (George, Ward-Robinson, & Pearce, 2001; Haselgrove, George, & Pearce, 2005).

In any case, the results of the three ID-ED experiments reported here imply that geometric information acquired from spatial navigation is not impervious to the influence of non-geometric information. With appropriate modification that acknowledges the role of learning on attention, associative analyses of spatial learning will provide an explanation of these experiments, as well as a reconciliation of extant conflicting findings. What might be more challenging for these theories, however, is an explanation for variations in the recognition of the shape of a navigated arena from a novel viewpoint.

## Supplementary Material

10.1037/a0034901.supp

## Complete Stage 1 and Stage 2 Counterbalancing Details

**Table:**

Group	Stage 1	Stage 2	Arena corners
Shape–Shape			
	K1	T1	
	K1	T2	
	K2	T3	
	K2	T4	
	K3	T2	
	K3	T4	
	K4	T1	
	K4	T3	
Landmark–Landmark			
	B1	R1	
	B1	R3	
	B2	R2	
	B2	R4	
	B3	R1	
	B3	R3	
	B4	R2	
	B4	R4	
Landmark–Shape			
	B1	T4	
	B1	T1	
	B2	T2	
	B2	T3	
	B3	T4	
	B3	T1	
	B4	T2	
	B4	T3	
Shape–Landmark			
	K1	R1	
	K1	R3	
	K2	R2	
	K2	R4	
	K3	R1	
	K3	R3	
	K4	R2	
	K4	R4	
*Note*. K = corners of the kite; T = corners of the trapezium; B = blue landmarks present in the kite; R = red landmarks present in the trapezium.			
