# Supplementary material for: Shape Shifting: Local Landmarks Interfere With Navigation by, and Recognition of, Global Shape
Source: J Exp Psychol Learn Mem Cogn. 2013 Nov 18;40(2):492–510. doi: 10.1037/a0034901 (PMC3933217; doi:10.1037/a0034901)
Supplement: Supplementary file 1 [file Buckley-Supplementary-Materials.docx]

Supplementary Materials

Experiment 1

Results

Figure S1 shows the mean distances traversed, in virtual units, from the beginning of each trial to enter the region defined as the hidden goal for the four groups during the 24 trials of stage 1 of the experiment. Similar to the latency data, mean distances traversed in the four groups decreased across this stage of the experiment. A two-way analysis of variance (ANOVA) of individual distances traversed, with the variables of relevant cue in stage one (landmarks or shape) and trial (1-24) revealed a significant main effect of trial, *F*(23, 1058) = 55.09, *MSE* = 597.81, but no main effect of relevant cue *F*<1. The interaction between Trial and Relevant cue was on the threshold of conventional significance *F*(23, 1058) = 1.59, *MSE* = 597.81, *p* = .05. Simple main effects analysis revealed that landmark relevant groups traversed shorter distances to find the goal on trials 1 and 4, but that the shape relevant group traversed shorter distances on trial 3. However, no significant differences in performance were noted by the end of stage 1.

***Figure S1 about here***

The mean distance traversed, in virtual units, to find the goal during stage 2 are shown in the top panel of Figure S2 for groups Shape-Shape and Landmark-Shape, and in the bottom panel of Figure S2 for groups Landmark-Landmark and Shape-Landmark. It can be seen that both groups that performed an ED shift (Groups Landmark-Shape and Shape-Landmark) showed longer latencies to find the goal relative to the appropriate ID groups (Groups Shape-Shape and Landmark-Landmark). A three-way ANOVA of individual latencies to find the goal, with the variables of shift (ID or ED), relevant cue in stage two (shape or landmarks), and trial (1-24), revealed a significant main effect of trial *F*(23, 1012) = 6.59, *MSE* = 540.56, and shift *F*(1, 44) = 59.42, *MSE* = 1784.20, but no main effect of relevant cue *F*<1. The interaction between Shift and Relevant cue was not significant *F*(1, 44) = 3.60, *MSE* = 1784.20. The interaction between Trial and Relevant cue was significant *F*(23, 1012) = 2.15, *MSE* = 540.56. Simple main effects analysis revealed that the ED groups were travelled more distance during the 24 trials of stage 2 than the ID groups *F*s(1, 44) > 16.88, *MSEs* = 1784.20, but there were no differences between the two ID groups, or between the two ED groups *F*s (1, 44) < 2.78, *MSE* = 1784.20. Importantly, the interaction between Shift and Trial was significant *F*(23, 1012) = 1.76, *MSE* = 540.56. Simple main effects analysis revealed that the ED groups were travelled a greater distance to find the goal on trials 2-10, 12-13, 15, 17, 19, 21-22, and 24 *Fs*(1, 1056) > 4.45, *MSEs* = 592.38.

***Figure S2 about here***

Experiment 2

Results

Figure S3 shows the mean distance traversed, in virtual units, of the 4 groups to find the hidden goal during the 24 trials of stage 1. In keeping with the results of Experiment 1, learning progressed at a similar rate in the four groups and the asymptotes of performance were similar. A two-way ANOVA of individual latencies to find the goal, with the variables of relevant cue in stage one (landmarks or shape) and trial (1-24), revealed a significant main effect of trial, *F*(23, 690) = 23.92, *MSE* = 477.35. There was no main effect of relevant cue, and no significant interaction between Relevant cue and Trial, *Fs*<1.

***Figure S3 about here***

The mean distances traversed, in virtual units, to find the goal during stage 2 are shown in the top panel of Figure S4 for groups Shape-Shape and Landmark-Shape, and in the bottom panel of Figure S4 for groups Landmark-Landmark and Shape-Landmark. In keeping with the results of Experiment 1, both groups that performed an ED shift (Groups Landmark-Shape and Shape-Landmark) showed longer latencies to find the goal relative to the appropriate ID groups (Groups Shape-Shape and Landmark-Landmark respectively). A three way ANOVA of individual latencies to find the goal, with the variables of shift (ID or ED), relevant cue in stage two (shape or landmarks) and trial (1-24), revealed a significant main effect of trial *F*(23, 644) = 9.45, *MSE* = 259.87, and of shift *F*(1, 28) = 16.88, *MSE* = 1987.56, but no main effect of relevant cue *F*(1, 28) = 2.62, *MSE* = 1987.56. The interaction between Shift and Relevant cue was not significant *F*(1, 28) = 1.06, *MSE* = 1987.56, nor was the interaction between Relevant cue and Trial *F*(23, 644) = 1.51, *MSE* = 259.87. Importantly, the interaction between Shift and Trial was significant *F*(23, 644) = 2.70, *MSE* = 259.87. Simple main effects analysis revealed that the ED groups traversed greater distances to find the goal that ID groups in trials 2-7, 9, and 15. The three-way interaction was not significant *F*(23, 644) = 1.07, *MSE* = 259.87.

***Figure S4 about here***

Experiment 3

Results

Figure S5 shows the distance traversed to find the hidden goal, in virtual units, during the 24 trials of stage 1 in the four groups. All groups showed a reduction in the distances traversed to find the goal as trials progressed, although it appeared that the groups for which landmarks were relevant may have found the goal quicker early in training. A two-way ANOVA of individual distances traversed to find the goal, with variables of relevant cue in stage one (landmarks or shape) and trial (1-24), revealed significant main effects of trial *F*(23, 2162) = 92.14, *MSE* = 312.54, but not relevant cue *F*(1, 94) = 3.56, *MSE* = 1321.48. The interaction between Trial and Relevant cue was also significant *F*(23, 2162) = 3.62, *MSE* = 312.54. Simple main effects analysis of the interaction revealed that groups for which landmarks were relevant traversed shorter distances to find the goal on trials 1 and 4 only *Fs*(1, 94) > 6.68, *MSEs* < 2946.45.

***Figure S5 about here***

The mean distances traversed, in virtual units, to find the goal during stage 2 are shown in the top panel of Figure S6 or groups Shape-Shape and Landmark-Shape, and in the bottom panel of Figure S6 for groups Landmark-Landmark and Shape-Landmark. In keeping with the results of Experiments 1 and 2, both groups that performed an ED shift (Groups Landmark-Shape and Shape-Landmark) showed longer latencies to find the goal relative to the appropriate ID groups (Groups Shape-Shape and Landmark-Landmark, respectively). A three-way ANOVA of individual latencies to find the goal, with the variables of shift (ID or ED), relevant cue in stage 2 (shape or landmarks), and trial (1-24) revealed significant main effects of trial *F*(23, 2116) = 15.24, *MSE* = 352.13, and shift *F*(1, 92) = 54.73, *MSE* = 2242.10, but not relevant cue *F*<1. There was no interaction between Shift and Relevant cue *F*(1, 92) = 2.93, *MSE* = 2242.10, or between Trial and Relevant cue *F*<1. There was, however, a significant interaction between Shift and Trial *F*(23, 2116) = 4.32, *MSE* = 352.13. Simple main effects analysis revealed that the ED groups traversed significantly greater distances to find the goal on trials 2-19, and 21-24 *Fs*(1, 92) > 6.03, *MSEs <* 782.20. Finally, returning to the results of the ANOVA, The three-way interaction was not significant *F*(23, 2116) = 1.45, *MSE* = 352.13.

***Figure S6 about here***

Figure Legends for supplementary materials

Figure S1: Mean distances traversed of the four groups to find the hidden goal in stage 1 of Experiment 1. Error bars show 1 +/- standard error of the mean.

Figure S2: *Top Panel.* Mean distances traversed of Groups Shape-Shape and Landmark-Shape to find the hidden goal in stage 2 of Experiment 1. *Bottom Panel.* Mean distances traversed of Groups Shape-Landmark and Landmark-Landmark to find the hidden goal in stage 2 of Experiment 1. Error bars show 1 +/- standard error of the mean.

Figure S3: Mean distances traversed of the four groups to find the hidden goal in stage 1 of Experiment 2. Error bars show 1 +/- standard error of the mean.

Figure S4: *Top Panel.* Mean distances traversed of Groups Shape-Shape and Landmark-Shape to find the hidden goal in stage 2 of Experiment 2. *Bottom Panel.* Mean distances traversed of Groups Shape-Landmark and Landmark-Landmark to find the hidden goal in stage 2 of Experiment 2. Error bars show 1 +/- standard error of the mean.

Figure S5: Mean distances traversed of the four groups to find the hidden goal in stage 1 of Experiment 3. Error bars show 1 +/- standard error of the mean.

Figure S6: *Top Panel.* Mean distances traversed of Groups Shape-Shape and Landmark-Shape to find the hidden goal in stage 2 of Experiment 3. *Bottom Panel.* Mean distances traversed of Groups Shape-Landmark and Landmark-Landmark to find the hidden goal in stage 2 of Experiment 3. Error bars show 1 +/- standard error of the mean

Figure S1

Figure S2

Figure S3

Figure S4

Figure S5

Figure S6
